# Molecular mechanisms of fat deposition: *IL-6* is a hub gene in fat lipolysis, comparing thin-tailed with fat-tailed sheep breeds

**DOI:** 10.5194/aab-64-53-2021

**Published:** 2021-02-17

**Authors:** Sana Farhadi, Jalil Shodja Ghias, Karim Hasanpur, Seyed Abolghasem Mohammadi, Esmaeil Ebrahimie

**Affiliations:** 1 Department of Animal Science, Faculty of Agriculture, University of Tabriz, Tabriz, Iran; 2 School of Animal and Veterinary Sciences, The University of Adelaide, South Australia 5371, Australia; 3 School of BioSciences, The University of Melbourne, Melbourne, Australia; 4 Genomics Research Platform, School of Life Sciences, La Trobe University, Melbourne, Victoria 3086, Australia

## Abstract

Tail fat content affects meat quality and varies significantly among different breeds of sheep. Ghezel (fat-tailed) and Zel (thin-tailed) are two important Iranian local sheep breeds with different patterns of fat storage. The current study presents the transcriptome characterization of tail fat using RNA sequencing in order to get a better comprehension of the molecular mechanism of lipid storage in the two mentioned sheep breeds. Seven (Zel = 4 and Ghezel = 3) 7-month-old male lambs were used for this experiment. The results of sequencing were analyzed with bioinformatics methods, including differentially expressed genes (DEGs) identification, functional enrichment analysis, structural classification of proteins, protein–protein interaction (PPI) and network and module analyses. Some of the DEGs, such as *LIPG*, *SAA1*, *SOCS3*, *HIF-1* α, and especially *IL-6*, had a close association with lipid metabolism. Furthermore, functional enrichment analysis revealed pathways associated with fat deposition, including “fatty acid metabolism”, “fatty acid biosynthesis” and “*HIF-1* signaling pathway”. The structural classification of proteins showed that major down-regulated DEGs in the Zel (thin-tailed) breed were classified under transporter class and that most of them belonged to the solute carrier transporter (SLC) families. In addition, DEGs under the transcription factor class with an important role in lipolysis were up-regulated in the Zel (thin-tailed) breed. Also, network analysis revealed that *IL-6* and *JUNB* were hub genes for up-regulated PPI networks, and *HMGCS1*, *VPS35* and *VPS26A* were hub genes for down-regulated PPI networks. Among the up-regulated DEGs, the *IL-6* gene seems to play an important role in lipolysis of tail fat in thin-tailed sheep breeds via various pathways such as tumor necrosis factor (TNF) signaling and mitogen-activated protein kinase (MAPK) signaling pathways. Due to the probable role of the *IL-6* gene in fat lipolysis and also due to the strong interaction of *IL-6* with the other up-regulated DEGs, it seems that *IL-6* accelerates the degradation of lipids in tail fat cells.

17 February 2021

## 1 Introduction

The development of adipose tissue has an effect on its function. Adipose tissue first appears around mid-gestation. Then, total adipose mass increases over the final few weeks of gestation (Symonds et al., 2012). In this time, it comprises a combination of brown and white adipocytes to enable the newborn to effectively adapt to the thermal and nutritional challenges of life after birth. Then, during postnatal life some, but not all, storage is replaced by white adipose tissue (Symonds, 2013).

The formation of adipose tissue in humans occurs with the growth of small and larger adipocytes and slow storage of fat within the cell until the age of 6–12 months postnatally. Small cells in the early stages of fat storage have an important role in fat mass and lipid storage in the first 6–12 months of life and are associated with increased fat cell size (Boulton et al., 1978).

Sheep are the main livestock producers of meat, milk and wool in the world, with nearly 25 % of the sheep population in the world being fat-tailed sheep (Zhou et al., 2017). The main factor influencing meat quality is the level of lipid storage in the carcass. Fat deposition efficiency in the tails of fat-tailed sheep is considerably higher than the other components of the carcass (Braissant et al., 1996; Li et al., 2018).

The most important component of the Iranian livestock industry is sheep production, which constitutes a total of nearly 50 million heads including more than 28 native sheep breeds which have great divergence in tail types (Moradi et al., 2012). Zel is the only thin-tailed breed (average 11 cm tail length), whereas the remaining breeds are considered fat-tailed (Valizadeh, 2010). The Zel (thin-tailed) breed is concentrated near the Caspian Sea, with almost 3 % of the Iranian sheep population (Vatankhah et al., 2006; Kamalzadeh et al., 2008). The Ghezel, in contrast, is a fat-tailed breed in the northwest of Iran, which possesses a large fat tail (Nabavi et al., 2014).

The tail fat is considered as an adaptive response of sheep to a hazardous environment and is a rich energy reservoir for the animal in winter and during migration when feed is scarce (Atti et al., 2004). It was also traditionally utilized by human beings as a preserver to save cooked meat for a longer period of storage time and also as an energy-rich food resource. Therefore, climatic changes as well as the associated requirements of human beings led to selection for heavier fat-tailed sheep across many generations (Atti et al., 2004; Kashan et al., 2005; Ermias et al., 2002; Gokdal et al., 2004). In spite of the previous efforts and natural forces that have led to the creation of heavy, fat-tailed breeds, it is not considered as a good characteristic in modern sheep production. Now, it is believed that the heavier tail decreases feed efficiency and that more energy is needed to deposit fat in the tail than to produce an equivalent amount of other tissue. The emphasis is on channeling the nutrients into leaner carcass generation to diminish the costs of fat deposition (Moradi et al., 2012; Bolger et al., 2014). Also, the market demand and monetary value of the fat-tailed breeds have been reduced and the tail fat has lost much of its advantage (Moradi et al., 2012). Furthermore, excessive fat in human daily diets causes some health problems. Therefore, a smaller fat tail is now desirable for both producers and consumers (Nejati-Javaremi et al., 2007). Prevention or reduction of tail fat deposition while enhancing lean meat production is the main purpose of the sheep industry. This has been eventually reached by docking at birth the fat tail, slaughtering at an early age, or by crossing the fat-tailed breeds with the thin-tailed breeds (Pourlis, 2011). The diversity of adipocyte volume across breeds is influenced by diet and genetic effects. The genetic effects are considered the main determinants for the formation and structure of adipocytes (Cheng et al., 2016; Davidson, 2014). Therefore, none of the aforementioned methods have been able to significantly reduce the tail fat in fat-tailed sheep.

To date, there have been several studies on the lipid metabolism of tail in different sheep breeds. Miao and Luo (2013) analyzed the transcriptome information of subcutaneous adipose tissue between Small-tailed Han and Dorset sheep. Wang et al. (2014) studied the diversity in the transcriptome profile of tail fat tissue between fat-tailed Kazak sheep and Tibetan short-tailed sheep and identified two top genes (*NELL1* and *FMO3*) with the largest expression differences between the two groups (Wang et al., 2014). Li et al. (2018) investigated the transcriptome of three adipose tissues from Guangling Large-tailed and Small-tailed Han sheep and reported four highly expressed co-genes, *FABP4*, *ADIPOQ*, *FABP5*, and *CD36* which are known as genes with close relation to adipose deposition (Li et al., 2018). Moreover, Bakhtiarizadeh et al. (2019) reported several enriched functional terms which might contribute to the deposition of fat in the tail of sheep via comparative transcriptome analysis between fat-tailed (Lori-Bakhtiari) and thin-tailed (Zel) Iranian sheep breeds (Bakhtiarizadeh et al., 2019). As mentioned, there were only a handful of high throughput, comprehensive studies that aimed to decipher the functional genes that differentiate the fat storage pattern of thin- and fat-tailed sheep breeds. Therefore, the current work was a further attempt to discover the functional genes that are responsible for fat deposition or fat lipolysis in the tail fat of sheep.

Whole transcriptome sequencing is a strong tool for exploring the genetic structure of complex traits (Wickramasinghe et al., 2014). Gene expression patterns may describe phenotypic differences in the tail fat of sheep breeds. Thus, the identification of gene networks and metabolic pathways involved in fat deposition or fat lipolysis could help to improve the quality of sheep meat and carcass (Bernard et al., 2007; Damon et al., 2013).

Knowledge about the molecular mechanism of fat storage is crucial for decreasing the fat mass in the carcass (Li et al., 2018). The mechanism of lipid metabolism is complex, and the manipulation of fat storage for lean meat production is very important in the sheep-breeding industry. The present study aimed to study the genetic profiles of fat tissues and to discover the diversity in the genetic mechanisms defining fat deposition between two morphologically different sheep breeds. We characterized the transcriptome of tail fat tissue from Ghezel (fat-tailed) and Zel (thin-tailed) sheep breeds. Analysis of the diversity of fat deposition between these two sheep breeds may aid the recognition of genes and pathways responsible for the formation of tail fat; therefore manipulation of fat deposition can contribute to new breeding strategies. We emphasized the DEGs and pathways involved in lipid metabolism and interpreted how they led to the differences in adiposity between Ghezel (fat-tailed) and Zel (thin-tailed) sheep breeds.

## 2 Materials and methods

### 2.1 Ethics statement

This study was carried out in strict accordance with the recommendations in the Guide for the Care and Use of Laboratory Animals of the National Institutes of Health. The protocol was approved by the Committee on the Ethics of Animal Experiments of the University of Tabriz, Iran (protocol number: 20170415/39/44).

### 2.2 Animals and samples

Eight healthy male lambs were selected for the experiment; four from the Ghezel (fat-tailed) breed and four from the Zel (thin-tailed) breed. All lambs were weaned at 90 d of age and fostered under similar conditions at the research station of the Faculty of Agriculture, University of Tabriz, Tabriz, Iran (Khalatpoushan). The lambs were housed in individual pens and provided the same diet ad libitum for 120 d and were slaughtered at age 7 months. The adipose tissues in the tail were rapidly collected under sterile conditions, instantly frozen in liquid nitrogen, and stored at -80 ${}^{\circ }$C until total RNA isolation.

### 2.3 RNA extraction

Total RNA was isolated from the tail adipose tissues using a Trizol reagent according to the manufacturer's instructions (TaKaRa, USA). In essence, fat tissues were powdered using liquid nitrogen, homogenized and centrifuged at 12 000 g for 10 min at 4 ${}^{\circ }$C to remove the insoluble material. Following the elimination of the top fatty layer on the aqueous phase, a clear supernatant was used for the next RNA extraction step. NanoDrop (Thermo Scientific NanoDrop 2000) was used to check the quantity of total RNA, and the 28 S / 18 S ratio was evaluated by electrophoresis on 1 % agarose gel to detect the RNA integrity. Finally, RNA samples that had a 28 s / 18 s ratio greater than 1 and an OD 260 nm / OD 280 nm ratio more than 1.9 were selected for sequencing. The integrity and concentration of RNA were measured using the 2100 Bioanalyzer (Agilent Technologies, Waldronn, Germany). The RNA integrity number value of all samples was above 7.0.

### 2.4 RNA sequencing and library preparation

Library preparation started with DNase I treatment, then poly(A) enrichment using oligo (dT) magnetic beads (Invitrogen, USA). The fragmentation step was conducted with short mRNA fragments which were subsequently used as templates for the first-strand cDNA synthesis. For the second-strand cDNA production, short fragments were ligated to adaptors with added poly(A) tails. After agarose gel electrophoresis, suitable fragments for PCR amplification were selected as templates. Unfortunately, one of the Ghezel (fat-tailed) samples failed in the library preparation step. Therefore, only seven paired-end cDNA libraries (four from Zel (thin-tailed) and three from Ghezel (fat-tailed) breeds) were produced. Finally, the sequencing of the libraries for the generation of 150 b paired-end reads was carried out using the Illumina HiSeq2000 platform. The generated raw RNA-Seq data were deposited into a NCBI SRA database with BioProject accession number PRJNA598581.

### 2.5 Quality control, mapping and quantification

FastQC (v0.11.5) was used for quality control of raw sequencing reads (Andrews, 2010). Trimmomatic software (v0.35) was used to trim the raw reads with adapter contamination, as well as for reads with more than 10 % of unknown bases and for reads with more than 50 % of low-quality bases (quality value ≤10) (Bolger et al., 2014). Finally, reads longer than 90 bp with a minimum Phred score of 20 were kept for the analyses. The clean reads were mapped to the sheep reference genome v4.0 (ftp://ftp.ncbi.nlm.nih.gov/genomes/Ovis_aries, last access: June 2020) using Bowtie (v2.2.4) (Langmead and Salzberg, 2012), SAMtools (v1.3.1) (Li et al., 2009) and TopHat (v2.1.1) (Trapnell et al., 2009). Assembled reads by Cufflinks (v2.2.1) were annotated with the NCBI reference annotation (ftp://ftp.ncbi.nlm.nih.gov/genomes/Ovis_aries) (Trapnell et al., 2012). Using Cuffmerge, individual transcripts were merged to form a single transcript assembly, and using Cuffdiff (v2.2.1), the merged transcript was used for quantification and differential expression analysis.

### 2.6 Gene expression analysis

Fragments per kilobase of exon per million fragments mapped (FPKM) values were used for the gene expression analysis. The FPKM for gene abundances was normalized for the library and gene length by comparing to an annotated reference genome (Oar_v4.0) (Mortazavi et al., 2008).

Gene expression analysis in the Ghezel (fat-tailed) and Zel (thin-tailed) breeds was performed with Cuffdiff. All differentially expressed genes with a log2 fold change larger than 1.1 at q value < 0.05 were selected. To obtain the similarity profile of gene expression in biological replicates of two breeds, principal component analysis (PCA) was conducted using web-based ClustVis tools (https://biit.cs.ut.ee/clustvis/, last access: July 2020).

### 2.7 GO classification analysis and KEGG pathway

We carried out the functional enrichment analysis for up- and down-regulated DEGs, separately. Therefore, in order to identify three categories of gene ontology (GO) including the biological processes, cellular components and molecular functions terms, the DEGs were submitted to the Enrichr Database (https://amp.pharm.mssm.edu/Enrichr/, last access: August 2020). Kyoto Encyclopedia of Genes and Genomes pathway analysis (http://www.genome.jp/kegg/, last access: August 2020) was used for pathway analysis. The adjusted p value < 0.05 was considered as the cutoff threshold for GO and KEGG pathway analyses.

### 2.8 Protein–protein interaction network and module analysis

To gain a further comprehension of the biological relationships between genes, the DEG list was inputted into the STRING database (https://string-db.org/, last access: September 2020). To define the functional modules, the constructed networks were clustered with the K mean algorithm to the three modules. Also, Cytoscape plug-in cytoHubba (v 3.7.2) was applied to detect the hub genes with the maximal clique centrality (MCC) method.

### 2.9 Structural classification of proteins

To find the structural class of genes related to lipid storage, total DEGs were imported to pathway studio web mammal version 11.2 (https://www.pathwaystudio.com/, last access: August 2020).

### 2.10 Validation of data

To confirm the different genes expressed, we retrieved the data of a similar study from the Gene Expression Omnibus (GEO) (https://www.ncbi.nlm.nih.gov/geo/, last access: October 2020) database, and gene expression analyses were conducted as above. Finally, the results were compared with our results to confirm the results of the current experiment.

## 3 Results

### 3.1 Sequencing data and mapping summary

A total of ∼47 gigabases containing 318 081 210 paired-end raw reads were generated. We obtained approximately 45 million paired-end clean reads of 150 bp for each sample and high percentages of mapped reads ranging from 82.40 % to 87.10 %. A mean of 85.17 % of clean reads was mapped to the sheep genome sequence (*Ovis aries* v4.0). Clean reads included 6.5 %–10.4 % non-uniquely mapped reads and 12.9 %–17.6 % unmapped reads. The summary of the mapping of samples is presented in Table 1.

**Figure 1:**
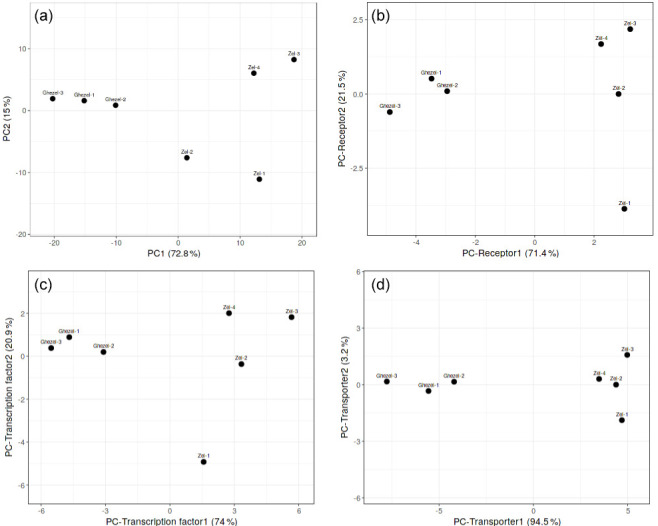
PCA scatter plot of differentially expressed genes in adipose tissues between Zel (thin-tailed) and Ghezel (fat-tailed) sheep breeds: **(a)** total differentially expressed genes, **(b)** differentially expressed receptors, **(c)** differentially expressed transcription factors, **(d)** differentially expressed transporters.

At first, PCA analysis of gene expression in the Zel (thin-tailed) and Ghezel (fat-tailed) breeds was performed to show the variance of the biological replicates of each breed. PCA results showed the distinct difference between the transcriptome profiles of Zel (thin-tailed) and Ghezel (fat-tailed) breeds (Fig. 1). PC analysis was also performed for the structural protein class of DEGs, and the transporter class showed better diversity between two breeds.

**Table 1:** Summary of RNA-seq data and mapping statistics of adipose samples of Zel (thin-tailed) and Ghezel (fat-tailed) sheep breeds.

Samples	Zel-1	Zel-2	Zel-3	Zel-4	Ghezel-1	Ghezel-2	Ghezel-3
Total reads	47 919 054	43 909 932	42 883 384	43 330 598	43 767 392	45 224 298	51 046 552
Total bases	7 187 858 100	6 586 489 800	6 432 507 600	6 499 589 700	6 565 108 800	6 783 644 700	7 656 982 800
Total mapped reads	37 339 712	35 103 330	33 763 526	34 616 084	35 112 150	34 757 959	39 097 097
	(84.3 %)	(87.1 %)	(85.5 %)	(86.7 %)	(86.8 %)	(82.4 %)	(83.4 %)
Non-uniquely mapped reads	5 389 716	2 323 331	2 212 652	2 297 504	2 371 892	2 880 142	4 049 800
Unmapped reads	10 579 342	8 806 602	9 119 858	8 714 514	8 655 242	10 466 339	11 949 455

According to the expression values, genes were classified into three groups including low expression (≤10 FPKM), moderate expression (10<FPKM≤500) and high expression (>500 FPKM). Transcriptome results showed that, in both breeds, most of the genes belonged to the low expression class (Table 2).

**Table 2:** Distribution of genes of different samples of Zel (thin-tailed) and Ghezel (fat-tailed) sheep breeds in three expression categories (high, moderate and low).

Samples	Zel (thin-tailed)				Ghezel (fat-tailed)		
	Zel-1	Zel-2	Zel-3	Zel-4	Ghezel-1	Ghezel-2	Ghezel-3
High expressed genes (>500 FPKM)	503	450	690	635	493	508	488
Moderate expressed genes (10<FPKM≤500)	6398	7196	9923	8436	7214	6637	6088
Low expressed genes (≤10 FPKM)	16 164	18 121	17 459	18 652	18 900	15 801	15 862
Total expressed genes	23 065	25 767	28 072	27 723	26 607	22 946	22 438
Total unexpressed genes	11 287	11 239	14 767	13 674	11 800	11 084	11 224

### 3.2 Analysis of some highly expressed genes

**Figure 2:**
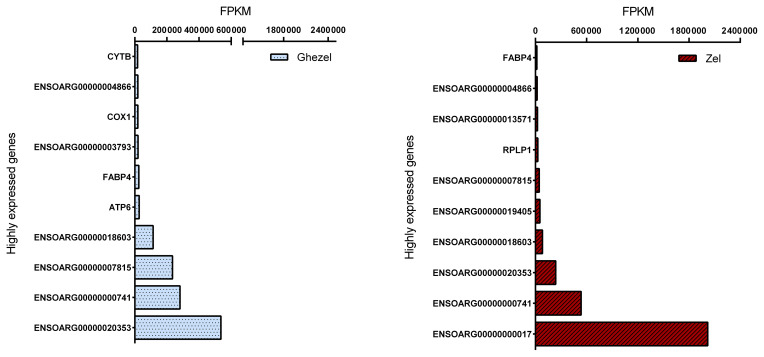
Top 10 highly expressed genes in adipose tissue of Ghezel (fat-tailed) and Zel (thin-tailed) breeds based on FPKM value.

Mapped reads abundances were normalized for library size and gene length (FPKM) to determine unbiased gene expression. Figure 2 shows the top 10 highly expressed genes (> 500 FPKM) for the Ghezel (fat-tailed) and Zel (thin-tailed) breeds. For both breeds, the top 10 highly expressed genes contributed to approximately 87 % of the total reads, which means that a small number of genes possessed a big portion of the total RNA of adipose tissue.

### 3.3 Expression level of differentially expressed genes in Ghezel (fat-tailed) and Zel (thin-tailed) breeds

**Figure 3:**
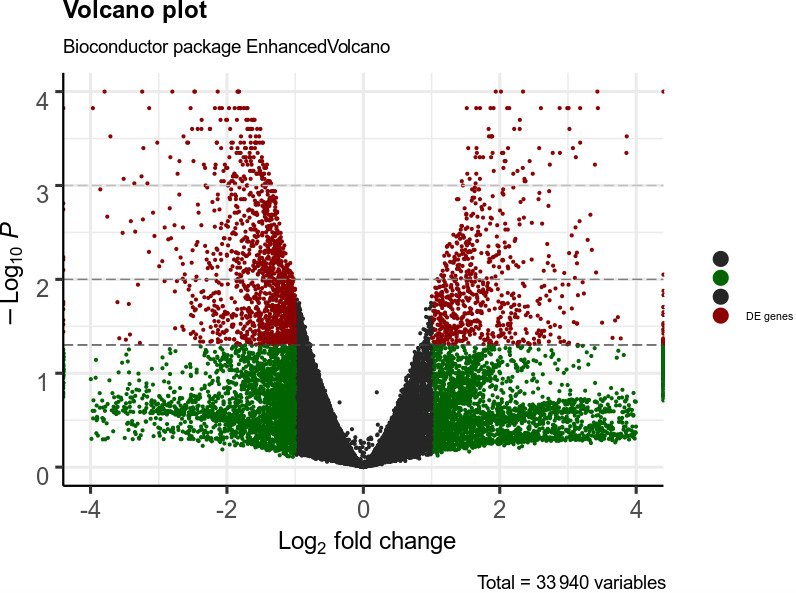
Volcano plot of expressed genes of adipose tissue of Zel (thin-tailed) compared to Ghezel (fat-tailed) sheep breeds. The y axis illustrates $-{\mathrm{log}}_{10}$ p values, and the x axis corresponds to a ${\mathrm{log}}_{2}$-fold change of gene expression between Zel and Ghezel sheep breeds. The red points represent significant genes (p value < 0.05).

**Figure 4:**
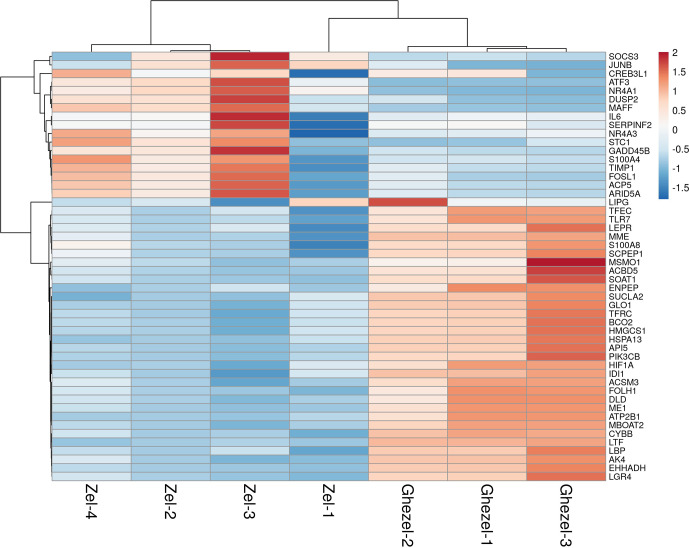
Expression profile of the 50 top differentially expressed genes in adipose tissue between Zel (thin-tailed) compared to Ghezel (fat-tailed) sheep breeds. Up- and down-regulated genes are separately colored with red and blue.

A total of 332 genes were found to be differentially expressed between Ghezel (fat-tailed) and Zel (thin-tailed). Out of the 332 DEGs, 78 were up-regulated, whereas the remaining 254 were down-regulated in the Zel (thin-tailed) breed. Total DEGs are presented in an additional spreadsheet file (see Table S1 in the Supplement). A volcano plot of the expressed genes is shown in Fig. 3. Also, the heatmap of the top 50 DEGs is shown in Fig. 4. A heatmap is a simple guide to assess the characteristics of RNA-seq results in which the various colors represent the level of differential expression. The red color represents a higher expression, and the blue color represents the lower expression.

The top 10 up- and down-regulated DEGs were sorted in Table 3.

**Table 3:** Top 10 up- and down-regulated genes in adipose tissue of Zel (thin-tailed) compared to Ghezel (fat-tailed) sheep breeds.

Ensemble gene ID	Gene name	Log${}_{2}$
		(fold change)
Up-regulated genes		
ENSOARP00000014659	*IL6*	6.98
ENSOARG00000017834	Unnamed	6.30
ENSOARG00000004007	*LIPG*	5.66
ENSOARG00000018812	*HSPB7*	5.42
ENSOARG00000019972	*SCG5*	3.86
ENSOARG00000009963	*SAA1*	3.62
ENSOARG00000010042	*STC1*	3.56
ENSOARG00000009119	*CCL19*	3.44
ENSOARG00000014064	*NR4A3*	3.39
ENSOARG00000000175	*SOCS3*	3.36
Down-regulated genes		
ENSOARG00000014064	Unnamed	13.52
ENSOARG00000020086	*SLC15A2*	6.09
ENSOARG00000008620	*LTF*	5.71
ENSOARG00000017865	*DNHD1*	5.10
ENSOARG00000020354	*MOGAT1*	4.89
ENSOARG00000018816	*LBP*	4.69
ENSOARG00000001217	*CYP4F21*	4.47
ENSOARG00000014789	*MBOAT2*	4.28
ENSOARG00000010196	*TNFRSF9*	3.97
ENSOARG00000003714	*ENPEP*	3.78

### 3.4 Functional enrichment analysis

Gene ontology analysis of DEGs was done for cellular components, molecular function, and biological process categories using the web-based Enrichr tool. The results were reported as significant in the case that adjusted p<0.05. The total terms that were enriched in the cellular components and molecular function category for total DEGs and biological processes for up- and down-regulated DEGs are presented in four supplemental spreadsheet files (see Tables S2, S3, S4 and S5, respectively).

**Figure 5:**
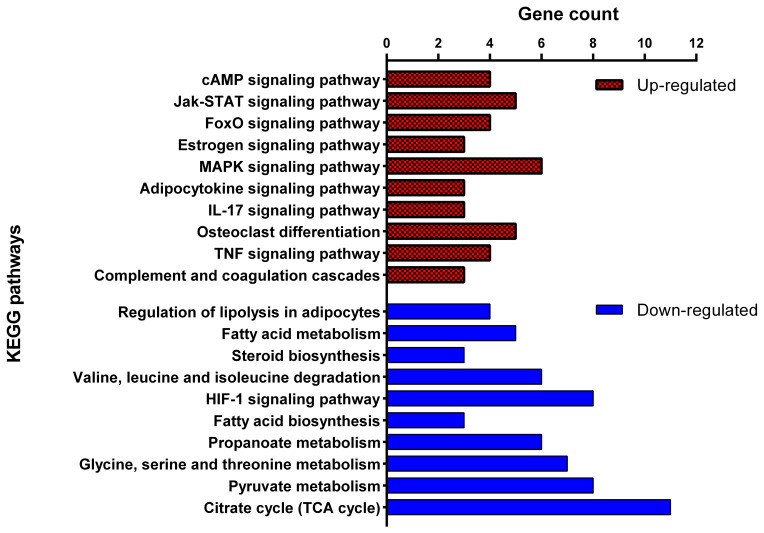
Top 10 significant Kyoto Encyclopedia of Genes and Genomes (KEGG) pathways enriched by up-regulated (red) and down-regulated (blue) differentially expressed genes of adipose tissue of Zel (thin-tailed) compared to Ghezel (fat-tailed) sheep breeds.

The cell component terms of “extracellular exosome”, “mitochondrion”, “peroxisome” and “extracellular matrix” as well as molecular function terms of “NAD binding”, “oligopeptide transporter activity” and “RNA polymerase II core promoter proximal region sequence-specific DNA binding” were significantly enriched with DEGs. Up- and down-regulated DEGs were mapped separately onto the KEGG pathway database to identify the related biological pathways with fat deposition (Fig. 5). For the 254 down-regulated DEGs in the Zel (thin-tailed) breed, 127 GO terms were significant (adjusted p<0.05), with most of the terms associated with fat metabolism. These are “monocarboxylic acid metabolic process”, “dicarboxylic acid metabolic process”, “acyl-CoA metabolic process”, “fatty acid metabolic process”, “cholesterol biosynthetic process”, “tricarboxylic acid metabolic process”, “glycerophospholipid biosynthetic process”, “membrane lipid metabolic process”, “fatty acid biosynthetic process”, “fatty-acyl-CoA metabolic process”, “fatty acid derivative biosynthetic process” and “long-chain fatty-acyl-CoA metabolic process”. The KEGG pathway analysis of down-regulated genes revealed 27 significant pathways (adjusted p<0.05) that had similar patterns to GO terms. These include “fatty acid biosynthesis”, “fatty acid metabolism”, “steroid biosynthesis”, “propionate metabolism”, and “pyruvate metabolism”. Furthermore, enrichment analysis of 78 up-regulated DEGs in the Zel (thin-tailed) breed showed 191 significant biological processes and 30 KEGG pathways that are related to fat metabolism. Some GO biological process terms for these genes such as “positive regulation of stress-activated mitogen-activated protein kinase (MAPK) cascade”, “activation of MAPKKK activity” and “positive regulation of p38MAPK cascade” are related to the “MAPK signaling pathway” which is activated during adipocyte lipolysis with rapid stimulation of hormone-sensitive lipase (HSL) activity.

### 3.5 Protein–protein interaction (PPI) network and module analysis

To reach a greater understanding of the biological relationships between the genes of a complex process such as fat deposition, the information about the functions of genes and proteins is required. Therefore, protein–protein interaction analysis is necessary. The up- and down-regulated DEGs were analyzed separately with the STRING database (v 10.5) (https://string-db.org/) (Szklarczyk et al., 2016). Of 332 DEGs, 300 were annotated in the STRING database and utilized to construct the PPI network. Enrichment analysis showed that PPI networks were significantly enriched (adjusted-p<0.05). Text mining, databases, gene fusion, co-expression, and neighborhood interactions were considered for the network construction. Disconnected nodes were deleted in the PPIs and scores of interaction confidence (confidence score < 0.4) were considered. To define the functional modules, the constructed networks were clustered with the K mean algorithm to the three modules. K mean is a common clustering algorithm that classifies a dataset into K (a predefined number) categories (Le and Kim, 2015). The Cytoscape plug-in cytoHubba (v 3.7.2) was applied to detect the hub genes with the MCC method. MCC has better accuracy for predicting principal proteins and hub genes in the PPI network (Chin et al., 2014; Chen et al., 2009). Hub genes have a main effect on the network topology (Albert and Barabási, 2002).

**Figure 6:**
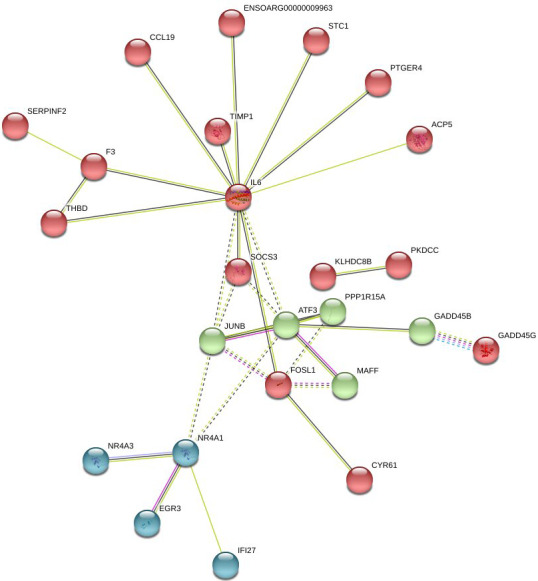
Protein–protein interaction (PPI) network and functional module analysis of up-regulated differentially expressed genes of adipose tissues of Zel (thin-tailed) compared to Ghezel (fat-tailed) sheep breeds.

In Fig. 6, the up-regulated DEGs in the red module (PPI enrichment p value: $3.92\times 10^{-11}$) included significant functional terms such as osteoclast differentiation (false discovery rate, FDR = 0.00060), insulin resistance (FDR = 0.0123), TNF signaling pathway (FDR = 0.0123), *IL-17* signaling pathway (FDR = 0.0123), complement and coagulation cascades (FDR = 0.0123), Jak-STAT signaling pathway (FDR = 0.0125), and cytokine–cytokine receptor interaction (FDR = 0.0200). DEGs in the blue module (PPI enrichment p value: $8.45\times 10^{-9}$) were included p53 signaling pathway (FDR = 0.00074), FoxO signaling pathway (FDR = 0.00074) and MAPK signaling pathway (FDR = 0.0016). *IL-6* and *JUNB* genes were also found as hub genes in the red and green module of up-regulated PPI, respectively.

**Figure 7:**
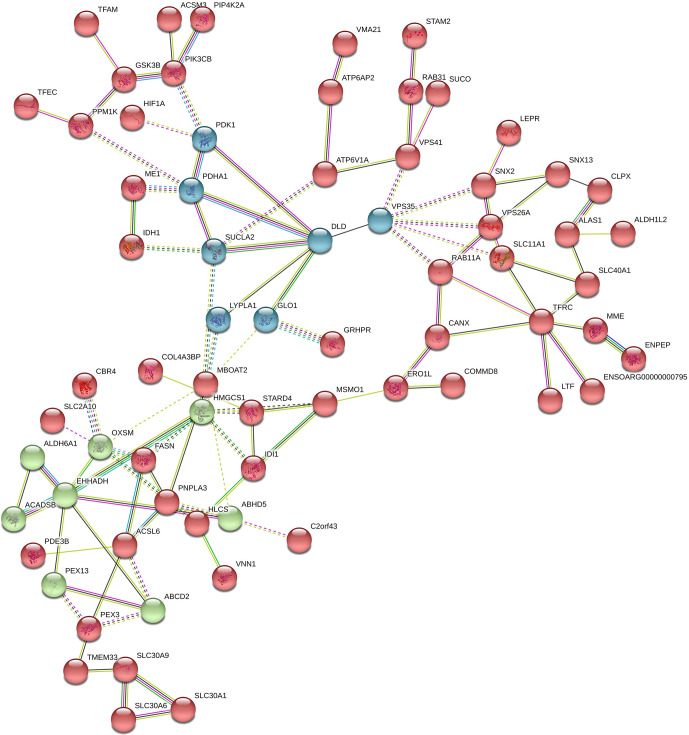
Protein–protein interaction (PPI) network and functional module analysis of down-regulated differentially expressed genes of adipose tissues of Zel (thin-tailed) compared to Ghezel (fat-tailed) sheep breeds.

In Fig. 7, the down-regulated DEGs in the green module (PPI enrichment p value: 0.0127) were significantly enriched in functional terms such as fatty acid biosynthesis (FDR = 0.00022), fatty acid metabolism (FDR = 0.00044), propionate metabolism (FDR = 0.00057) and pyruvate metabolism (FDR = 0.00057). Significant terms in the green module were more strongly associated with fat metabolism than the other modules. DEGs in the red module (PPI enrichment p value: $5.55\times 10^{-16}$) were related to glycine, serine and threonine metabolism (FDR = $9.58\times 10^{-5}$); pyruvate metabolism (FDR = 0.00049); glycolysis/gluconeogenesis (FDR = 0.0030); and HIF-1 signaling pathways (FDR = 0.0167), as well as biosynthesis of amino acids (FDR = 0.0246), biotin metabolism (FDR = 0.0257) and regulation of lipolysis in adipocytes (FDR = 0.0472). DEGs in the blue module (PPI enrichment p value: $1.0\times 10^{-16}$) were related to steroid biosynthesis (FDR = $1.25\times 10^{-11}$) and terpenoid backbone biosynthesis (FDR = 0.00021). Also, the *HMGCS1* gene was identified as a hub gene for the green module, *VPS35* as a hub gene for the blue module and *VPS26A* as a hub gene for the red module in down-regulated PPI.

### 3.6 Structural classification of proteins

Total DEGs were imported to a web-based pathway studio. Out of 332 DEGs, 107 were present in seven classes, including transporter, transcription factor, receptor, ligand, protein kinase, protein phosphatase and pseudogene. Expression patterns of the DEGs were mainly classified as genes encoding transporters, transcription factors and receptors, respectively. PCA results demonstrated that most of the DEGs in each of the aforementioned three clusters showed a distinct expression profile of tail fat in two sheep breeds (Fig. 1). Also, some of the annotated DEGs in the transcription factor class, such as *JUNB*, *NR4A3*, *HIF-1* α, *FOSL1*, *MAFF*, *NR4A1*, *CREB3L1*, and *ATF3* were related to lipolysis function. More interesting is that all aforementioned genes except the *HIF-1* α gene were up-regulated in the Zel (thin-tailed) breed. Gene expression is regulated by activating or suppressing transcription factors, so the aforementioned DEGs may have stimulated lipolysis of fat in the tail of Zel (thin-tailed) sheep.

### 3.7 Validation of differentially expressed genes

**Figure 8:**
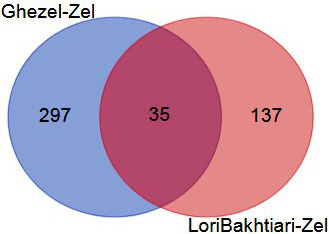
Venn diagram of the differentially expressed genes in the current study (Ghezel vs. Zel comparison) and Bakhtiarizadeh et al. (2019) study (Lori-Bakhtiari vs. Zel comparison).

**Figure 9:**
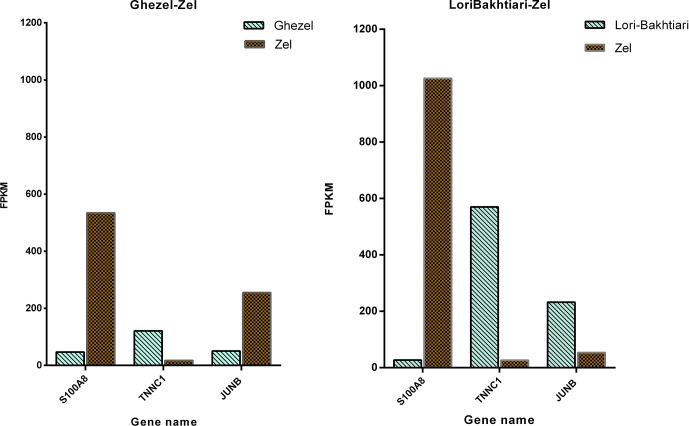
Gene expression levels of three differentially expressed genes in the current study and Bakhtiarizadeh et al. (2019) study. y axis illustrates the expression levels of three genes based on FPKM value.

To validate the DEGs of the current study we reanalyzed the data of the Bakhtiarizadeh et al. (2019) study. The Venn diagram of the DEGs showed 35 common DEGs between the two studies (Fig. 8). Of the 35 common genes between the two studies, 28 genes showed the same direction expression and 7 genes demonstrated opposite direction of expression. Their common genes and expression direction are presented in a supplemental spreadsheet file (see Table S6). Among the DEGs that were validated by q-RT-PCR in the study of Bakhtiarizadeh et al. (2019), we detected three common DEGs including *S100A8*, *TNNC1* and *JUNB* genes. Two out of those three genes showed the same expression patterns (Fig. 9).

## 4 Discussion

The mechanism of fat deposition is complex and the improvement of meat production is of great importance in sheep breeding. The main goal of the present study was to define the genetic mechanisms underlying lipid storage in the tail of Iranian sheep breeds. The comparison of two morphologically different sheep breeds was made with regard to the effect of breed on fat deposition. In our study, samples of tail fat tissues were collected at the end of adipocyte differentiation stage. So, the higher expression level in the adipocytes of the fat-tailed breed demonstrates the stronger role of these genes in adipose metabolism than in thin-tailed sheep breeds. Classification of proteins also showed that, out of 35 DEGs in the transporter class, 11 DEGs belonged to the SLC families. SLC transporters are the largest transporter family and responsible for transporting inorganic ions, amino acids and lipids across the membrane. All these genes were up-regulated in the Ghezel (fat-tailed) breed. Therefore, it can be postulated that the overexpression of SLC family genes in the Ghezel (fat-tailed) breed might contribute to the accumulation of fat in the tail.

Out of the top 10 highly expressed genes, 4 were in common in both breeds, including ENSOARG00000000741, ENSOARG00000007849, ENSOARG00000020353 and ENSOARG00000018603. It seems that these genes have an important, determinant role in the metabolism of adipose tissue. Furthermore, fatty acid binding protein 4 (*FABP4* = ENSOARG00000009344) and cytochrome c oxidase 1 (*COX1* = ENSOARG00000000016) genes may play an important role in the regulation of fat deposition in the Ghezel (fat-tailed) breed. *FABP4* is a well-known gene that is involved in lipid metabolism, and it has been demonstrated that its plasma level is progressively increased according to body mass index (BMI) (Queipo-Ortuño et al., 2012) and shows a positive correlation with body weight (r=0.377) (Auguet et al., 2014). Furthermore, recent studies have shown that *COX* pathways may be involved in both increased size and the number of mature adipocytes in the differentiation pre-adipocyte process. These results suggested that two isoforms of *COX* had different functions: with *COX2*, which is involved in body fat regulation, and *COX1*, whose role is not yet determined (Yan et al., 2003). As a result, although further research is needed to determine the effect of *COX1* and *FABP4* genes on the accumulation of fat in the tail, the higher expression of both *COX1* and *FABP4* genes in the Ghezel (fat-tailed) than in the Zel (thin-tailed) breed might suggest a potential role for them in the deposition of excessive fat in the tail of fat-tailed sheep breeds (Fig. 2).

Results of up- and down-regulated DEGs show that some down-regulated DEGs in the Zel (thin-tailed) breed such as *LTF*, *LBP*, *MOGAT1* and *MBOAT2* are related to lipid storage. Min et al. (2018) reported that lactoferrin (LTF) has favorable effects on body weight and lipid accumulation in mice. In another study the up-regulation of LTF has been observed in Small-tailed Han sheep as compared with that of Tibetan thin-tailed sheep (Ma et al., 2018). Up-regulation of LTF in the Ghezel (fat-tailed) breed suggests that this gene has a key function in fat deposition in fat-tailed sheep breeds. Another down-regulated DEG in the Zel (thin-tailed) breed is lipid-binding protein-5 (*LBP-5*) gene. As a result, the lipid-binding protein family is essential for homeostasis of fatty acid and lipid transfer (Moreno-Navarrete et al., 2017), and *LBP-5* regulates fat accumulation in *Caenorhabditis elegans* and is bound directly to fatty acids with varying affinities (Xu et al., 2011). Indeed, FABPs are members of the intracellular LBP that have a role in the accumulation of fat (Zheng et al., 2013). Similar to *LBP-5*, the expression of the monoacylglycerol O-acyltransferase 1 (*MOGAT1*) gene is inversely correlated with the lipolytic rate, and its suppression results in the enhancement of basal lipolytic activity (Liss et al., 2018; Shi et al., 2019). So this result suggests that the up-regulation of the aforementioned genes can make more fat deposition in the tail of the Ghezel (fat-tailed) breed (Table 3). Some up-regulated DEGs in the Zel (thin-tailed) breed are associated with fat lipolysis, such as *IL6*, *SAA1*, *LIPG*, *NR4A3* and *SOCS3* (Table 3). As a result, both inhibitions of lipolysis and the promotion of lipogenesis could lead to the increment of fat in the tail of Ghezel (fat-tailed) sheep. In contrast, the overexpression of the aforementioned genes in the thin-tailed breeds may result in a smaller tail. One of the important genes in the lipolysis of fatty acids is nuclear receptor subfamily 4 group A member 3 (*NR4A3*). There have been reports that the overexpression of NR4A3 produced a marked reduction in body weight in mice and several insulin-resistant rodent models (Walton et al., 2016). Similar to *NR4A3*, endothelial lipase (*LIPG*) is a form of lipase that is secreted by vascular endothelial cells and plays an important role in lipoprotein metabolism and the lipid composition of cells (Justine et al., 2018). Lipase characteristics of this gene suggest a significant pivotal degradation role for decreasing fat deposition. Another up-regulated gene in the Zel (thin-tailed) breed was serum amyloid A1 (*SAA1*), which has been reported to play a critical role in the β oxidation of lipids as well as in promoting lipolysis and inhibiting the expression of genes related to lipogenesis. *SAA1* inhibited the expression of perilipin which acts as a protective coating from the body's natural lipases, such as hormone-sensitive lipase (*HSL*) (Wang et al., 2010). Like other up-regulated DEGs, interleukin-6 (*IL-6*) participates in the metabolism of fat, which elevates lipolysis and the release of liver free fatty acids and triglycerides (Zhang et al., 2014). *IL-6* affects lipid metabolism and, being a lipolytic factor, stimulates the lipolysis and oxidation of fatty acids in humans (van Hall et al., 2003; Xu et al., 2018), bovines (Contreras et al., 2017b) (Contreras et al., 2017a), mice (Han et al., 2018; Ma et al., 2015) and rats (Nonogaki et al., 1995). But the role of *IL-6* in lipolysis of the fat in sheep has not yet been fully elucidated. Up-regulation of this gene may contribute to the lipolysis of fatty acids in the tail of the Zel (thin-tailed) breed. *IL-6* is, also, a hub gene in the red module of the PPI network of up-regulated genes (Fig. 6) with a pivotal interaction with other DEGs. These findings suggest an important role for aforementioned genes in decreasing fat deposition in the tail of the Zel (thin-tailed) breed. *IL-6* was also enriched in several significant GO terms including “positive regulation of cellular process” and “regulation of signaling receptor activity”.

Results of enrichment analysis revealed that several pathways are involved in lipolysis such as tumor necrosis factor (TNF) signaling and MAPK signaling pathways. *IL-6* gene is one of the important genes that enriched the mentioned pathways. MAPK is the main signal-regulating lipid metabolism. Activation of MAPK inhibits fat synthesis and promotes fatty acid oxidation (Grisouard et al., 2012). Interestingly, the *IL-6* gene has a strong role in MAPK signaling and TNF signaling pathways, because *IL-6* promotes lipid breakdown, glycolysis and fatty acid oxidation in skeletal muscle and adipose tissues via the MAPK signaling pathway. Ruderman et al. (2006) showed that *IL-6* is mainly found in adipose tissue and the center of the hypothalamus and regulates the body fat composition. The deletion of the *IL-6* gene in mice led to obesity and insulin resistance (Matthews et al., 2010; Duszka et al., 2013). In general, most of the pathways related to lipolysis have *IL-6* in common. Other enriched DEGs in the MAPK pathway such as nuclear receptor subfamily 4 group A member 1 (*NR4A1*) (Zhao et al., 2018), dual specificity phosphatase 1 (*DUSP1*) (Wu et al., 2015), growth arrest and DNA damage-inducible beta (*GADD45B*) (Kim et al., 2014), and growth arrest and DNA damage-inducible gamma (*GADD45G*) (Fuhrmeister et al., 2016) were associated with lipid metabolism. Up-regulation of these genes may result in an increased lipolysis in Zel (thin-tailed) sheep. One of the other KEGG signaling pathways that is enriched by *IL-6* is the TNF signaling pathway, which removes lipid droplets of perilipin and activates hormone-sensitive lipase (HSL) and adipose triglyceride lipase (ATGL). Both HSL and ATGL release free fatty acids by acting on triacylglycerol and diacylglycerol (Yang et al., 2011). Other up-regulated DEGs that are enriched in the TNF signaling pathway are CAMP responsive element binding protein 3 like 1 (*CEREBL1*), *JunB* proto-oncogene, AP-1 transcription factor subunit (*JUNB*) and suppressor of cytokine signaling (*SOCS3*), related to lipolysis of fat. The TNF signaling pathway is well-known to increase adipocyte lipolysis (Feingold et al., 1992; Green et al., 1994; Hauner et al., 1995; Kawakami et al., 1987). A recent study demonstrated that *SOCS3* plays a critical role in the regulation of fatty acid β oxidation and is an important factor for lipid metabolism (Luo et al., 2011). Interestingly, our findings showed a strong interaction between *IL-6* and *SOCS3* genes. The *CREB3L1* gene has also been reported to increase glycerol release and has significant effects on lipolysis by participating in the CAMP signaling pathway. According to Ehrlund et al. (2017), *CREB3L1* knockdown led to reduced glycerol release and had significant effects on lipolysis. Wang et al. (2016) investigated the involvement of *CREB3L1* in the maintenance of lipid homeostasis and found that CREB3L1 deletion leads to a defect in triglyceride (TG) lipolysis, resulting in higher levels of plasma TG. Up-regulation of these genes may enhance adipocyte lipolysis and increase the concentrations of circulating free fatty acids in the Zel (thin-tailed) breed. The results of the current work may suggest a crucial role for DEGs related to TNF signaling and MAPK signaling pathways, especially for *IL-6*, in increasing lipid lipolysis, and also in differentiating the thin-tailed sheep breeds from fat-tailed ones.

Finally, validation of three selected DEGs revealed that two of them (i.e. *TNNC1* and *S100A8*) had the same direction of expression in our study and the study of Bakhtiarizadeh et al. (2019). Results of the two studies show that *TNNC1* and *S100A8* genes had higher and lower expression in fat-and thin-tailed sheep breeds, respectively. Troponin C1, slow skeletal muscle and cardiac type (*TNNC1*) are related with the tenderness of meat (Wang et al., 2020). Also, an important paralog of this gene is troponin C2, fast skeletal type (*TNNC2*), which exists in the longissimus muscle area and is strongly related with fat deposition (Silva et al., 2019). *S100A8* is a well-known inflammatory-response related gene of obese adipose tissue that emphasize its essential role on the pathogenesis of obesity (Sekimoto et al., 2015). High circulating levels of *S100A8* in obese individuals have been confirmed in mice (Sekimoto et al., 2012) and human (Chen et al., 2020). But *S100A8* gene in two study were up-regulated in Zel (thin-tailed) breed. Unlike the above two genes, the *JUNB* gene had a different expression direction, and as mentioned above, this gene is enriched in the TNF signaling pathway, which is one of the important pathways for fat lipolysis. In addition, the *JUNB* gene in the green module of up-regulated genes as a hub gene may play a major role in fat lipolysis.

## 5 Conclusions

In the present study, we compared the gene expression profile of the Ghezel (fat-tailed) with that of the Zel (thin-tailed) sheep breed with focus on genes related to fat metabolism. Our results indicate that known fat metabolism pathways such as “fatty acid metabolism”, “fatty acid biosynthesis”, “pyruvate metabolism” and “HIF-1 signaling pathway” are more active in the deposition of fat in the tail of Ghezel (fat-tailed) sheep, whereas “TNF signaling pathway” and “MAPK signaling pathway” and related genes stimulate lipolysis in Zel (thin-tailed) sheep. Differential expression of some fat-metabolism-related genes such as *IL-6*, *NR4A1*, *SOCS3*, *ATF3*, *CREB3L1*, *HIF-1* α, *HMGCS1*, *JUNB*, *LIPG*, *NR4A3*, *FOSL1*, *VPS35*, *VPS26A*, *LTF*, *LBP* and *MBOAT2* might contribute to the genetic and morphologic diversity of sheep breeds. *IL-6*, *LIPG* and *SAA1* genes were associated with fat lipolysis and *LTF*, *LBP*, *MOGAT1* and *MBOAT2* were associated with fat deposition at a very high level of expression in the Zel (thin-tailed) and Ghezel (fat-tailed) breeds, respectively. Strong interaction between the up-regulated DEGs was shown via PPI network analysis. This implies that DEGs of these modules, especially the *IL-6* gene, are major candidate genes in the tail fat metabolism of sheep. Our findings also showed that enriched modules with significant KEGG pathway terms have a strong association with fat metabolism. In addition, the structural classification of proteins showed that the DEGs under the transcription factor class play an important role in the lipolysis of the Zel (thin-tailed) breed. Finally, our findings led to a further understanding of the distribution and regulation of fat deposition in different types of tails in sheep breeds.

## Supplement

10.5194/aab-64-53-2021-supplement The supplement related to this article is available online at: https://doi.org/10.5194/aab-64-53-2021-supplement.

## Data Availability

The generated raw RNA-Seq data were deposited into the NCBI SRA database with BioProject accession number PRJNA598581.
