# Supplementary material for: Molecular mechanisms of fat deposition: IL-6 is a hub gene in fat lipolysis, comparing thin-tailed with fat-tailed sheep breeds
Source: Arch Anim Breed. 2021 Feb 17;64(1):53–68. doi: 10.5194/aab-64-53-2021 (PMC8130542; doi:10.5194/aab-64-53-2021)
Supplement: The supplement related to this article is available online at: https://doi.org/10.5194/aab-64-53-2021-supplement. [file aab-64-53-supplement.zip › aab-64-53-2021-supplement-title-page.pdf]

Supplement of Arch. Anim. Breed., 64, 53–68, 2021
https://doi.org/10.5194/aab-64-53-2021-supplement
© Author(s) 2021. This work is distributed under
the Creative Commons Attribution 4.0 License.

Open Access
Archives Animal Breeding

Supplement of

Molecular mechanisms of fat deposition: *IL-6* is a hub gene in fat lipolysis, comparing thin-tailed with fat-tailed sheep breeds

Sana Farhadi et al.

Correspondence to: Sana Farhadi (farhadiso16@tabrizu.ac.ir)

- aab-64-53-2021-supplement-title-page.pdf
- Supplementary
  - S1.xlsx
  - S2.xlsx
  - S3.xlsx
  - S4.xlsx
  - S5.xlsx
  - S6.xlsx

The copyright of individual parts of the supplement might differ from the CC BY 4.0 License.
